# Supplementary material for: MOdulation-Guided ENcoding (MOGEN) Scheme for Vessel-Encoded Arterial Spin Labeling
Source: Magn Reson Med. Author manuscript; Available in PMC 2026 Jul 1. (PMC7618936; doi:10.1002/mrm.70335)
Supplement: Supplementary Information [file EMS213006-supplement-Supplementary_Information.pdf]

Supporting information

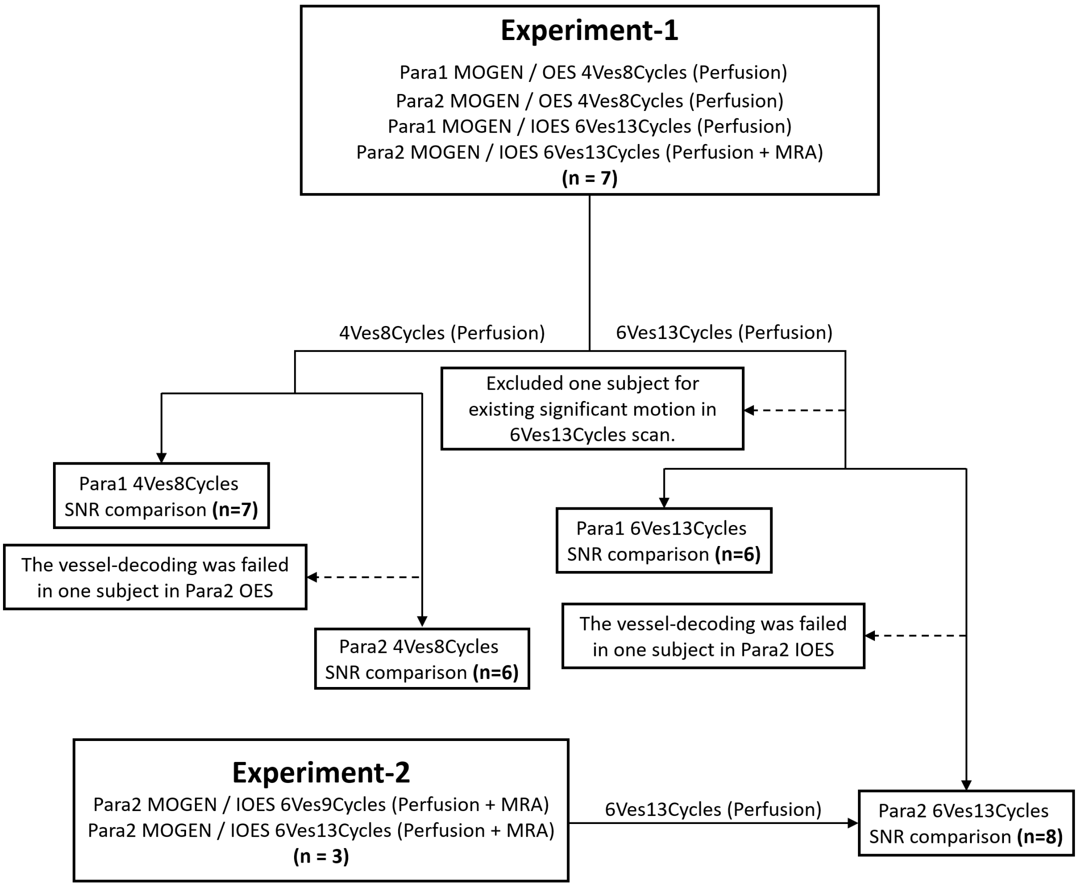

Figure S1. Flowchart detailing the subject inclusion process in the two experiments. Experiment 1: seven subjects were included, Para1 and Para2 were applied in 4Ves8Cycles and 6Ves13Cycles to compare the SNR between MOGEN and OES / IOES. One subject showed failed decoding in both OES and IOES with Para2, and thus was excluded from the statistical analysis. Experiment 2: three subjects were included for the six-vessel scenario with 6Ves9Cycles and 6Ves13Cycles using Para2. An additional scan of 2D dynamic vessel-encoded MRA was performed for the case with Para2 and six vessels. Statistical comparisons in SNR were performed only for the perfusion scans.

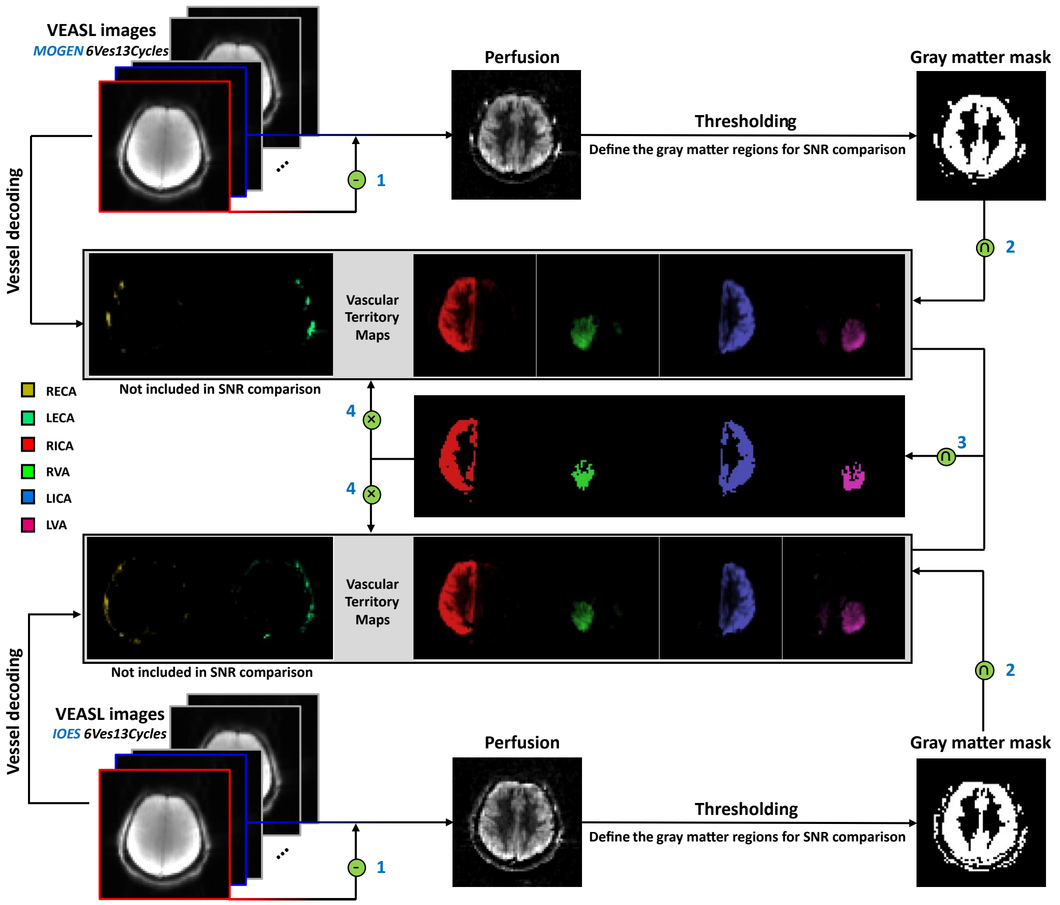

Figure S2. Procedure for calculating SNR of the in vivo VEASL perfusion data, exemplified with a 6Ves13Cycles scan. (1) The non-selective pairs were subtracted to generate perfusion maps and an empirical threshold were used to define the gray matter region; (2, 3) The decoded VTI images from MOGEN and IOES for each territory of ICAs and VAs were intersected with the gray matter to define the voxels with dominant supply for each artery; (4) The mean signal for each territory was extracted from the VTI images, weighted by the number of voxels in each territory, divided by the standard deviation of the background region, and then averaged to calculate the mean SNR.

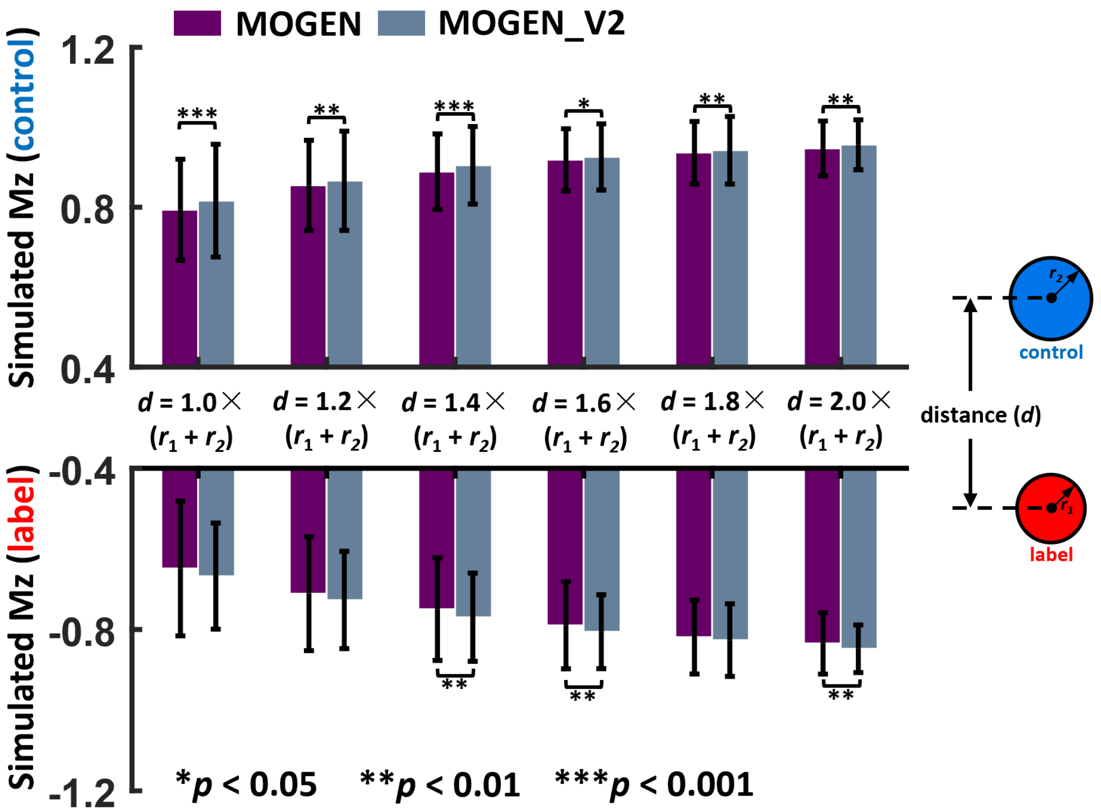

Figure S3. Accounting for vessel size via multi-voxel representation enabled MOGEN_V2 to achieve a better labeling effect than MOGEN when the vessels of interest were spatially close, which also revealed that increased inter-vessel distance was associated with a progressive rise in target labeling efficiency and enhanced suppression of adjacent control vessels.

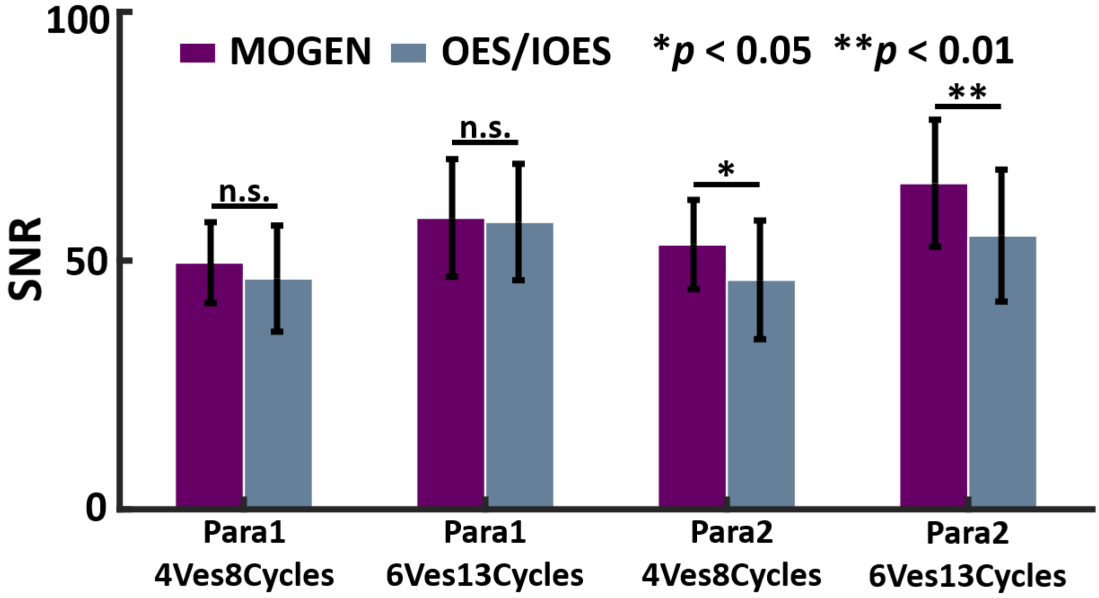

Figure S4. Comparison of mean SNR on healthy volunteers between MOGEN and OES/IOES under different PCASL parameters, number of vessels and number of encoding cycles. Using Para1, the SNR of MOGEN was higher than OES/IOES but not significant. Using Para2, the MOGEN method exhibited significantly higher SNR than OES/IOES.

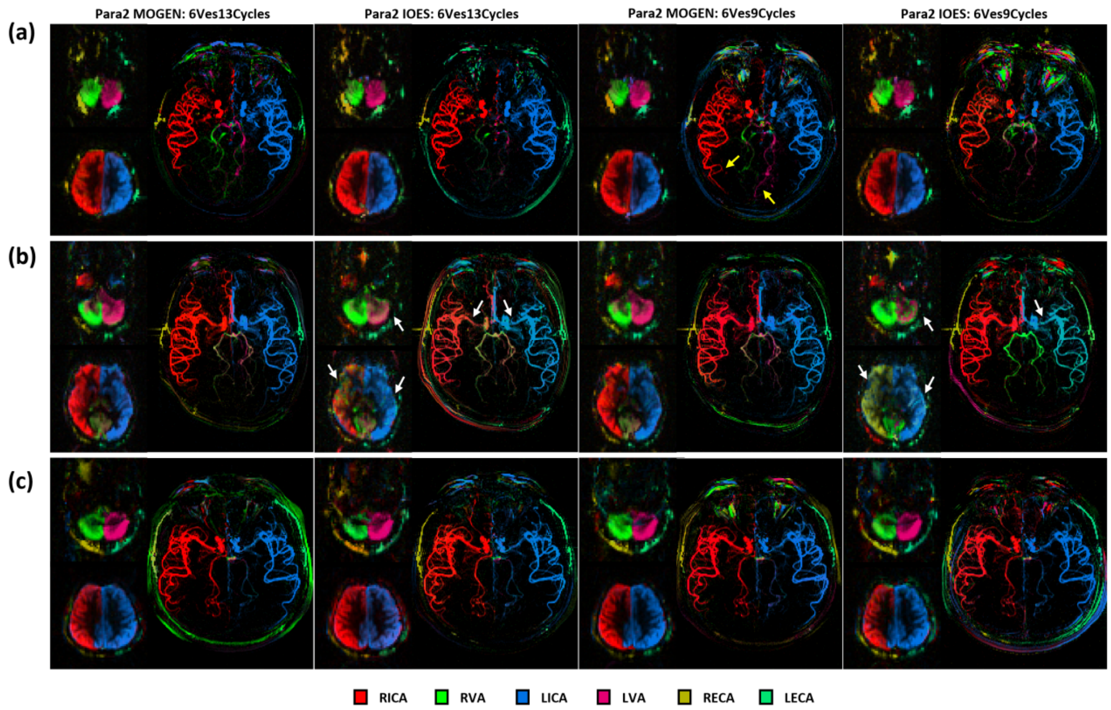

Figure S5. Comparison of the VTI maps between 13 and 9 cycles on three subjects from Experiment 2. For MOGEN, reducing cycles from 13 to 9 has little impact on vessel encoding. However, for IOES, as seen in the second row, more encoding cycles do contribute to improved encoding performance (white arrow). In the first row, MRA shows improved distal visualization in MOGEN with 6Ves9Cycles (yellow arrow), indicating that increasing the number of cycles does not necessarily enhance SNR efficiency in MOGEN.

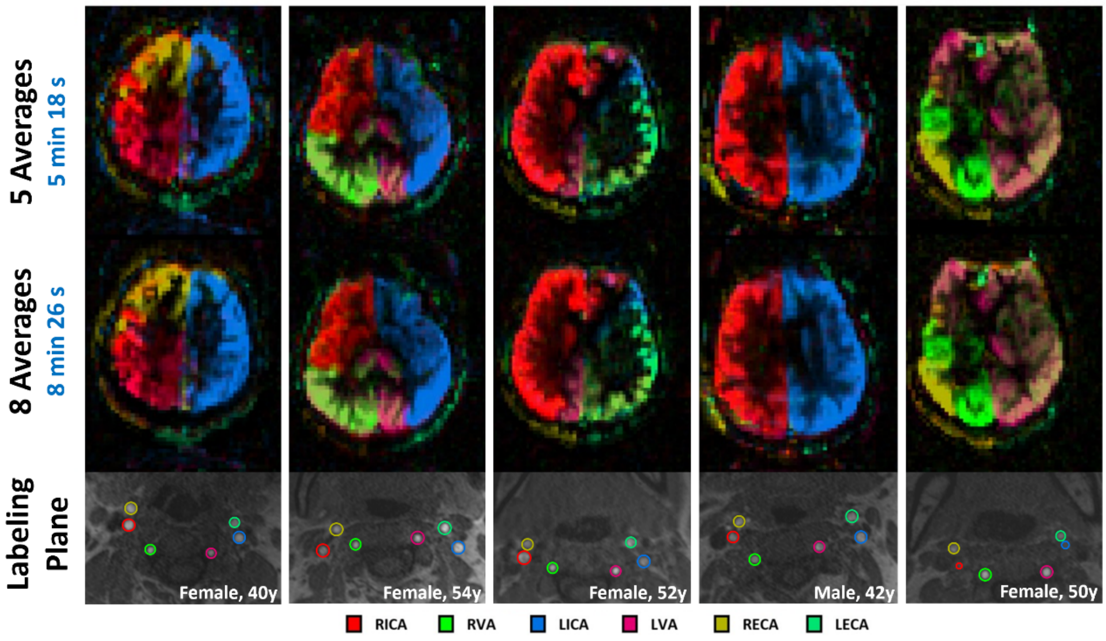

Figure S6. Typical VTI maps obtained with 5 averages and 8 averages using MOGEN from 5 MMD patients. When only 5 averages (with a total scan time of 5 min 18 s) were used, the decoding performance was generally similar to that with 8 averages (8 min 26 s), without a substantial compromise of the visualization of collateral pathways.

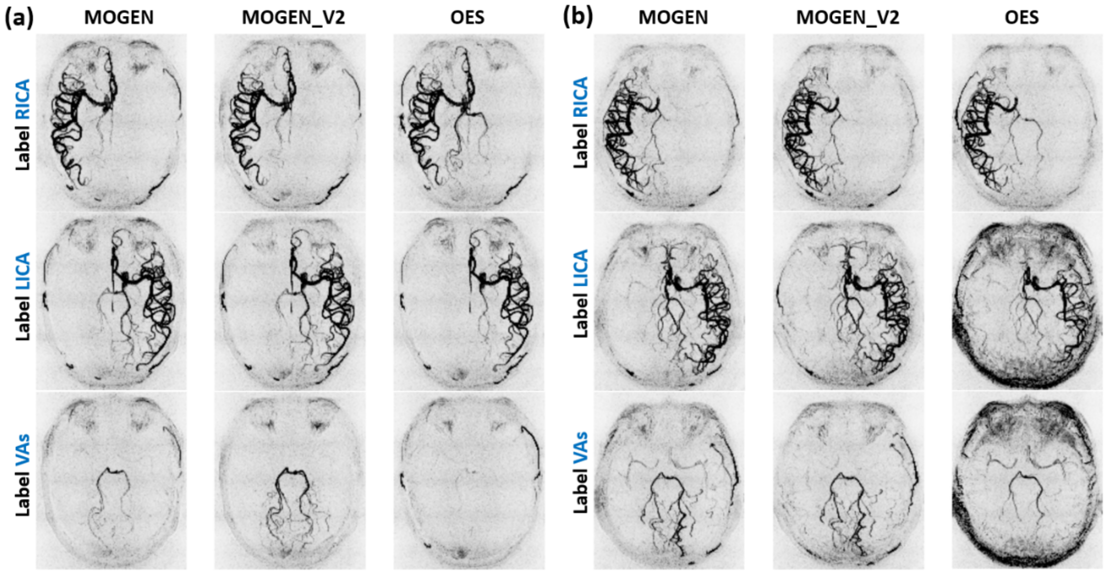

Figure S7 Representative vessel encoded MRA images from two healthy volunteers.

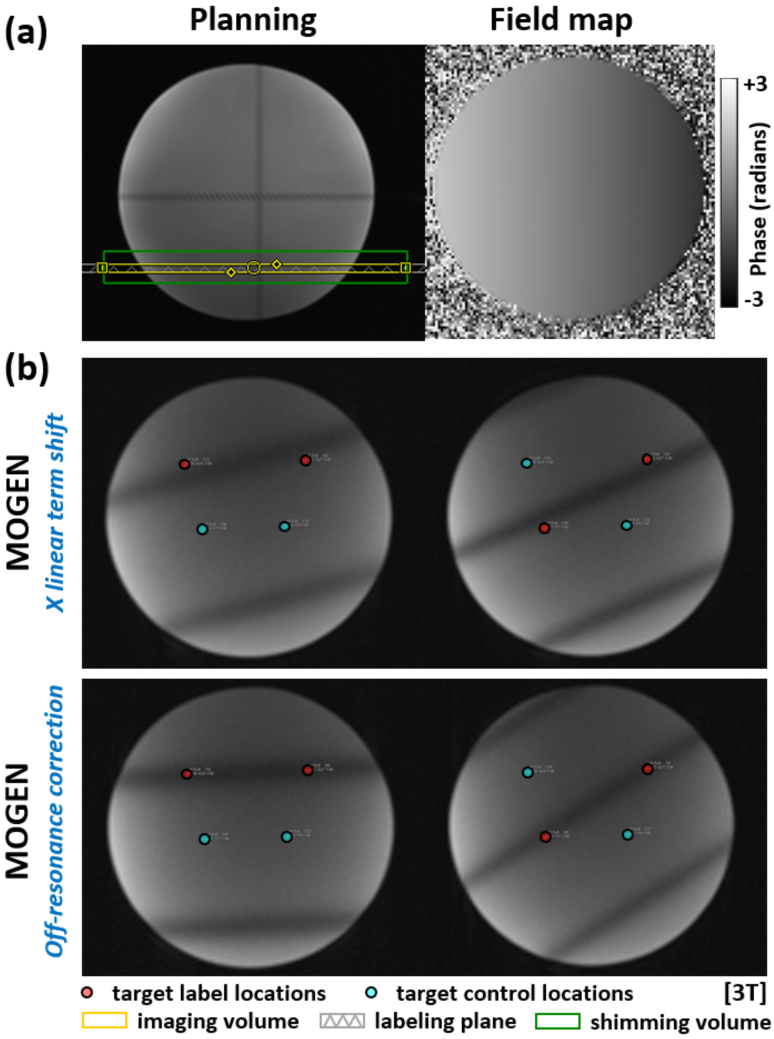

Figure S8. Illustration of the MOGEN-based off-resonance correction on phantom. (a) shows the locations of the labeling and imaging planes, as well as the measured field map. (b) shows two different encoding patterns before and after off-resonance correction. The encoding patterns were shifted due to off-resonance in the X direction and were corrected using the MOGEN-based off-resonance correction.

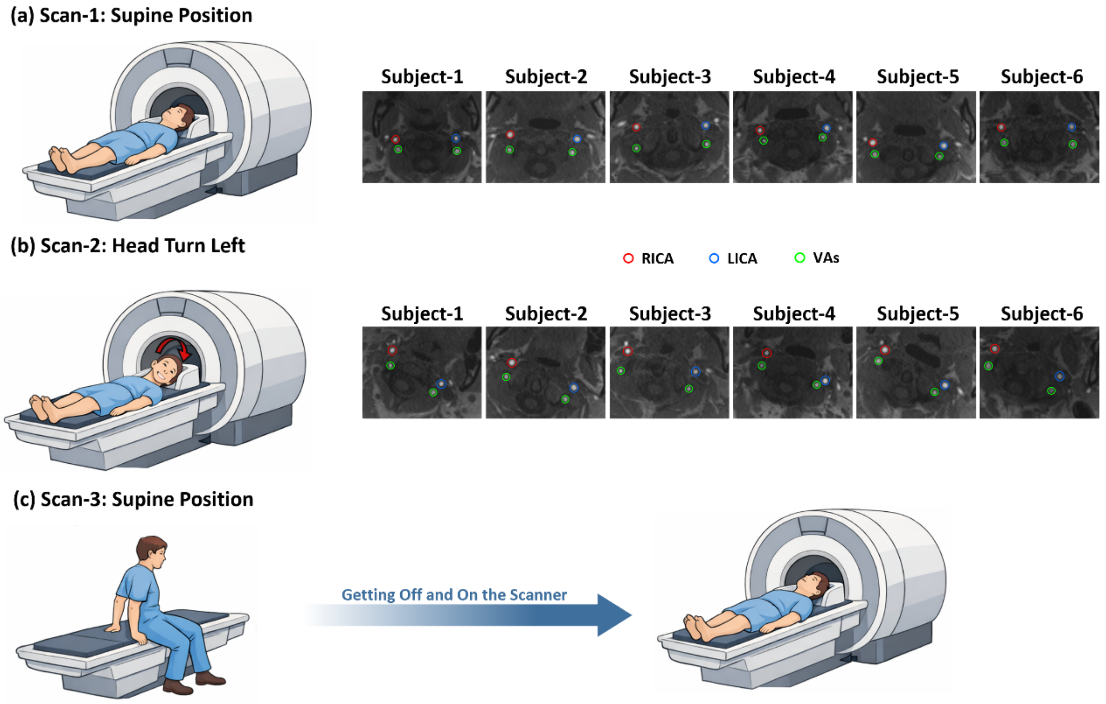

Figure S9. Design of the experiment for assessing robustness of the VEASL with MOGEN against variability of artery geometry. Each subject underwent three identical scanning sessions, consisting of a 28 s time-of-flight (TOF) scan for vascular localization, followed by MOGEN calculation and a 3 min 56 s VEASL scan (Para2, five encoding cycles, 10 averages). (a) First, the subject was scanned in a routine supine position; (b) the table was then moved out, and the subject rotated the head to the left by a large angle; (c) finally, the subject exited the scanner and re-entered it, and another scan was performed. The labeling planes for each subject in the first two scans are provided, showing changes of the artery locations. Abbreviations: RICA, right internal carotid artery; LICA, left internal carotid artery; VA, vertebral artery.

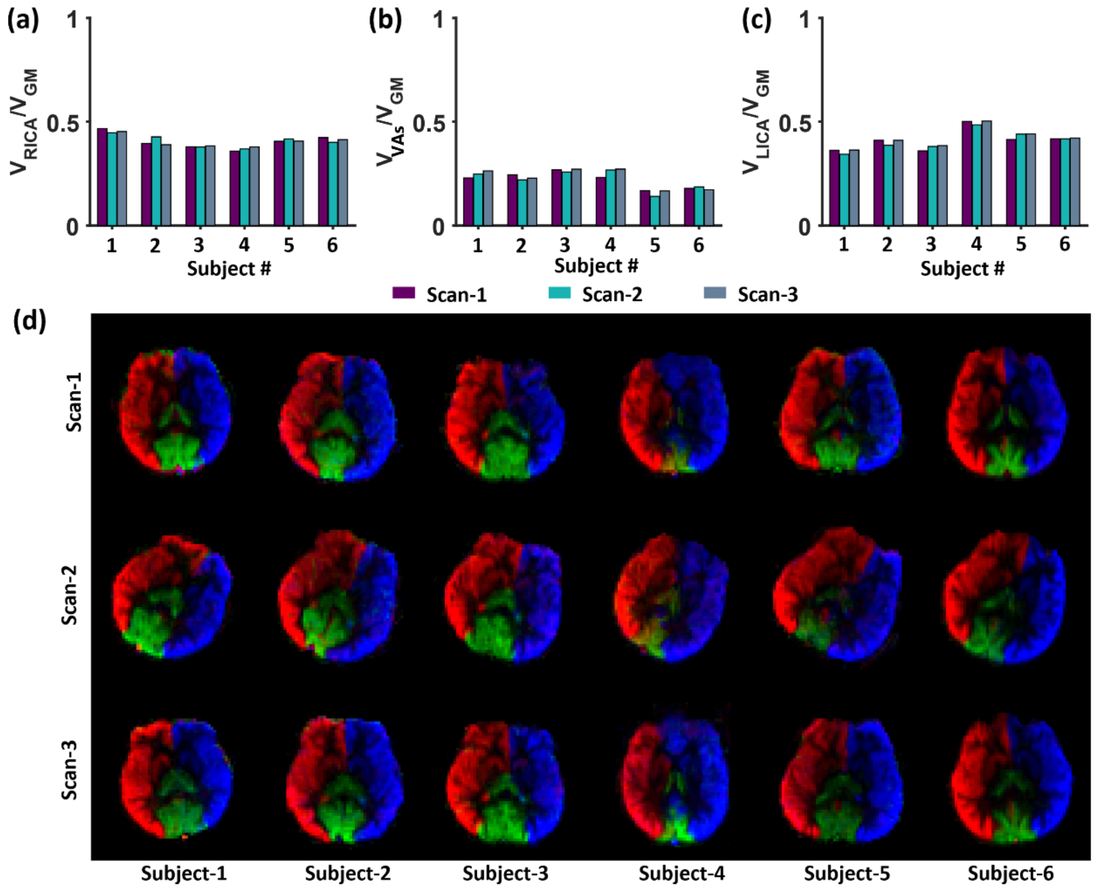

Figure S10 Vessel-decoding results of VEASL across the three scanning sessions. (a-c) Vascular perfusion territory fractions of the RICA, VAs, and LICA relative to gray matter volume for six subjects. (d) VTI maps for the six subjects. Abbreviations: RICA, right internal carotid artery; VA, vertebral artery; LICA, left internal carotid artery; GM, gray matter; VTI, vascular territory image.

Table S1. Measurement consistency of vascular perfusion territory fractions across three repeated scans in six subjects was assessed. A two-way random-effects intraclass correlation coefficient with absolute agreement for single measurements was used to quantify reliability.

Vascular perfusion territory fraction	Intra-class coefficients
V_{RICA}/V_{GM}	0.87 (95%CI [0.55;0.98], $P < 0.001$
V_{VAs}/V_{GM}	0.90 (95%CI [0.67;0.98], $P < 0.001$
V_{LICA}/V_{GM}	0.94 (95%CI [0.80;0.99], $P < 0.001$

Table S2. Presence of collateral flow assessed by DSA and VEASL in five Moyamoya patients.

	Vertebral arteries				External carotid arteries	
	RVA-R	RVA-L	LVA-R	LVA-L	RECA-C	LECA-C
P1	0/0	1/1	1/1	1/1	1/1	0/0
P2	1/1	0/0	1/1	1/1	0/0	0/0
P3	0/0	1/1	1/1	1/1	0/0	0/0
P4	1/1	0/0	1/1	0/0	0/0	×/0
P5	1/1	1/1	1/1	1/1	1/1	1/1

0 = no collateral flow detected; 1 = collateral flow detected. RVA-R and RVA-L indicate collateral flow from the right vertebral artery to the right and left anterior circulation, respectively; LVA-R and LVA-L denote collateral flow from the left vertebral artery to the right and left cerebral hemispheres, respectively. RECA-C and LECA-C indicate the presence or absence of collateral flow from the right and left external carotid arteries to the cerebrum, respectively. Left/right of the slash indicates DSA/VEASL assessment, respectively. “×” indicates missing DSA images for the corresponding artery.
